# Social Media Perspectives on Clavicle Fractures: A Content Analysis of Instagram Posts From Patients and Surgeons

**DOI:** 10.7759/cureus.79480

**Published:** 2025-02-22

**Authors:** Ryder Davidson, Kaitlyn Novotny, Raul Saucedo, Jalen Paulos, Karen Nelson, Janel Pietryga, Christopher Fang

**Affiliations:** 1 Department of Orthopedics, Kirk Kerkorian School of Medicine at the University of Nevada, Las Vegas (UNLV), Las Vegas, USA

**Keywords:** clavicle, fracture, instagram, patient perspective, social media

## Abstract

Introduction

Both clinicians and patients use social media platforms like Instagram to share images and insights about clavicle fractures, although the content and its alignment with clinical practice may vary. While most stable clavicle fractures are managed nonoperatively, social media posts may disproportionately highlight less common treatment options. This study aimed to evaluate the content and themes related to clavicle fractures shared by patients and surgeons on Instagram.

Methods

An Instagram search was conducted using the three most common hashtags related to clavicle fractures: #ClavicleFracture, #BrokenCollarbone, and #BrokenClavicle. A total of 1,500 posts from January 2021 to January 2024 were analyzed, consisting of the first 500 posts from each hashtag. Data collected for each post included the number of likes, comments, and followers, as well as image content, post type, treatment approach (operative vs. nonoperative), tone, and source type (surgeon vs. patient). Surgeon and patient posts were compared, while posts without a specified author were excluded.

Results

A total of 1,083 posts were included in the study, with 210 (20%) from surgeons and 873 (80%) from patients. Surgeons were more likely to use the hashtag #ClavicleFracture, whereas patients favored the other hashtags (p < 0.001). X-rays comprised 54% of surgeon posts, compared to 23% of patient posts. Patients were significantly more likely to share images of skin, slings, or reflective photos (p < 0.001). Surgeon posts were primarily educational, while patient posts tended to be more reflective. Most surgeon posts conveyed a positive tone, whereas patient posts were more likely to have a negative tone (p < 0.001). Regarding treatment depiction, 75% of surgeon posts featured operative treatment, whereas only 33% of patient posts did, with the majority (60%) not specifying their treatment outcome (p < 0.001).

Conclusions

The content and language used in Instagram posts about clavicle fractures differ between surgeons and patients. Surgeons are more likely to use technical jargon, share X-rays, provide educational content, adopt a positive tone, and focus on operative treatment. However, their posts generally receive lower social media engagement compared to those by patients.

## Introduction

Social media encompasses internet-based platforms that connect individuals, organizations, and communities, enabling them to share and consume multimedia - such as text, images, videos, and diagrams - on topics ranging from personal experiences to geopolitics and medical advancements. It is an increasingly integral part of modern society. Instagram alone boasts over 1.4 billion users worldwide, a number projected to reach 1.8 billion by 2028 [1]. With social media’s rapid and sustained growth over the past decade, more people are turning to these platforms to seek and share health information [2,3]. This shift has the potential to enhance public health literacy and empower patients to make informed decisions about their care. However, our understanding of the complex relationship between social media, patients, clinicians, and medical knowledge is still in its early stages, requiring further investigation.

Epidemiological studies indicate that clavicle fractures are common, accounting for 2-10% of all fractures [4]. These injuries affect both men and women across all age groups, with an average age of 29 at the time of fracture [5,6]. Given their high incidence, their prevalence among individuals under 30 - a demographic known for significant social media engagement - and their often visible cosmetic impact, clavicle fractures provide a relevant case study for exploring how health information is shared and perceived by both patients and surgeons on social media [7].

Historically, most clavicle fractures were managed nonoperatively. However, recent evidence suggesting lower nonunion rates and faster return to function has led to a shift toward surgical intervention [6,8]. This study aims to analyze differences in perspectives and content between surgeons and patients regarding clavicle fractures by examining Instagram posts. We hypothesize that physician posts will primarily emphasize operative management and adopt a more positive tone, whereas patient posts will focus more on the injury itself and convey a reflective perspective. Understanding these differences could help improve preoperative discussions, enhance public health literacy, and refine shared decision-making between patients and healthcare providers.

## Materials and methods

A single search was conducted on February 28, 2024 using the three most commonly used hashtags for clavicle fractures on Instagram (Meta Platforms, Inc., Menlo Park, CA, USA): #BrokenCollarbone, #ClavicleFracture, and #BrokenClavicle. The first 500 posts from each hashtag, as presented by Instagram’s algorithm, were collected, resulting in a total of 1,500 posts. To prevent overrepresentation, if a single user had more than five unique posts within any category, only their first five posts were included in the analysis. Posts were excluded if the identity of the poster was unspecified or if the account was private. The dataset included posts published between January 2021 and January 2024.

Data collection encompassed several variables, including the number of likes, picture content, poster’s follower count, theme, treatment type, tone, and source. Statistical analyses were performed using SAS Studio (SAS Institute, Cary, NC, USA) to compare differences between surgeon and patient posts. Chi-square tests were used to assess differences in categorical variables, while Student’s t-tests analyzed differences in continuous quantitative variables. Statistical significance was set at p < 0.05. As this study exclusively utilized publicly available information, it was exempt from institutional review board approval.

## Results

Post demographics

Out of the 1,500 posts collected, 1,083 posts met the inclusion criteria, comprising 210 (20%) posts by surgeons and 873 (80%) by patients (Table 1). Posts from other categories (e.g., parents/guardians, medical organizations) were excluded (417 posts). Surgeons were more likely to use the hashtag #ClavicleFracture (90%) compared to patients (17%), while patients primarily employed the hashtags #BrokenCollarbone (44%) and #BrokenClavicle (39%) (p < 0.001). In addition, patient posts received more likes (91 vs. 65; p < 0.001) and featured a greater number of total posts on their profiles (987 vs. 416; p < 0.001). Although surgeon accounts had a higher average number of followers than patient accounts (5,842 vs. 3,038), this difference was not statistically significant (p = 0.18) (Table 2).

**Table 1:** Summary of hashtag use on Instagram, categorized by author

Hashtag	Surgeons		Patients		p-Value
	Number	Percentage	Number	Percentage	
#BrokenCollarbone	13	6%	382	44%	<0.001
#ClavicleFracture	189	90%	152	17%	<0.001
#BrokenClavicle	8	4%	339	39%	<0.001
Total posts	210		873		<0.001

**Table 2:** Demographics of Instagram accounts with posts regarding clavicle fractures

Variable	Surgeons		Patients		p-Value
	Mean	Range	Mean	Range	
Likes	65	0-1,184	91	0-3,953	<0.001
Posts	416	2-3,785	987	2-41,891	<0.001
Followers	5,842	8-404,000	3,038	1-117,343	0.18

Post content

The majority of surgeon posts featured radiographs (54%), whereas radiographic images appeared in only 23% of patient posts (p < 0.001) (Table 3). Patients were more likely than surgeons to share images of skin (10% vs. 1%; p < 0.001), slings (16% vs. 1%; p < 0.001), and other reflective content (32% vs. 20%; p < 0.001).

**Table 3:** Overview of clavicle fracture post content on Instagram by author

Variable		Surgeons		Patients		p-Value
		Number	Percentage	Number	Percentage	
Picture content	X-ray	113	54%	203	23%	<0.001
	Skin	3	1%	84	10%	<0.001
	Surgery	5	2%	1	0.10%	<0.001
	Rehab	1	0.50%	49	6%	<0.001
	Sling	2	1%	138	16%	<0.001
	Two or more	43	21%	122	14%	<0.001
	Other	43	20%	276	32%	<0.001
	Total posts	210		873		
Post type	Educational	126	60%	4	0.46%	<0.001
	Reflection	80	38.10%	636	72.85%	<0.001
	Other	4	1.90%	233	26.69%	<0.001
	Total posts	210		873		
Tone	Positive	138	65.71%	384	43.99%	<0.001
	Negative	10	4.76%	215	24.63%	<0.001
	Unspecified	62	29.52%	274	31.39%	<0.001
	Total posts	210		873		

In terms of content, surgeon posts were predominantly educational (60% vs. 0.5%; p < 0.001), while patient posts were more often reflective (73% vs. 38%; p < 0.001). A positive tone was common in both groups, although more prevalent in surgeon posts (66% vs. 44%; p < 0.001). However, negative-tone posts appeared significantly more often among patients than surgeons (25% vs. 5%; p < 0.001).

Operative vs. nonoperative treatment

**Figure 1:**
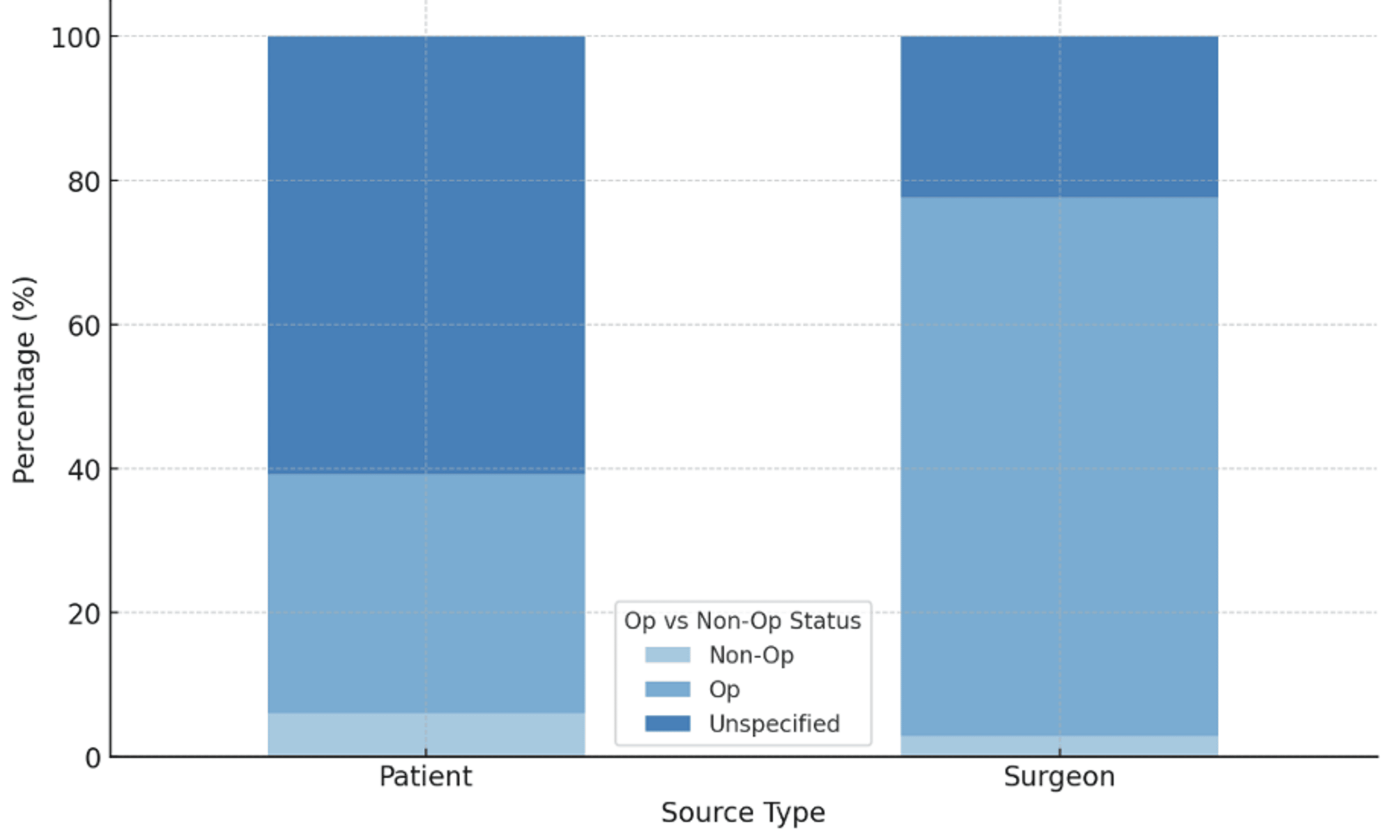
Instagram posts categorized by author, showing the treatment modalities for clavicle fractures

Posts by surgeons more frequently depicted operative treatment than those by patients (75% vs. 33%; p < 0.001) (Figure 1). Notably, 60% of patient posts did not specify their mode of treatment.

## Discussion

The increasing use of social media, particularly as a platform for sharing and consuming unregulated medical information, has the potential to be either a challenge or an advantage for the medical community in the years ahead. It offers physicians a direct and innovative means of medical outreach and education, potentially improving patient outcomes, communication, expectations, and overall patient-centered care. Despite its rapid and widespread adoption, social media remains in its early stages when it comes to its role in medicine. To fully harness its benefits, it is essential to understand how both patients and surgeons are currently utilizing these platforms. This study aimed to explore these dynamics by examining the differences in social media posts from surgeons and patients regarding a common medical condition: clavicle fractures.

Our analysis of 1,083 posts revealed that the majority (80%) were shared by patients. Additionally, patient posts tended to receive more likes and had a higher number of followers compared to those created by surgeons. In contrast, a 2023 study that used similar methods to examine posts about spinal cord stimulation found that surgeons contributed more posts than patients (39% vs. 24%) [9]. This discrepancy may stem from the relative commonality of clavicle fractures compared to the specialized and less frequently performed spinal cord stimulation procedure. The latter likely appeals to a more surgeon-focused audience, which could explain the differing trends in post distribution.

Furthermore, incorporating hashtags with more specialized medical terminology, such as #ClavicleSurgery or #ClavicleORIF, might yield a dataset with a different demographic composition. A notable gap persists between patients and surgeons in their use of medical versus everyday terminology, including fundamental distinctions such as “fracture” versus “broken”. Future research should explore the most commonly used terms by both groups when discussing basic medical conditions.

Our data revealed a notable contrast in the content of posts made by providers compared to those by patients. Surgeons predominantly highlighted their operative treatment processes, whereas patients focused on rehabilitation and quality of life. Patient posts were often personal narratives, recounting the initial injury or describing their recovery journey. They also tended to include images of healing skin or the use of a sling. In contrast, surgeons typically shared educational content, often incorporating radiographs in their posts.

These findings are consistent with previous research on social media engagement regarding medical issues. For example, a study by Juliebo-Jones et al. examined posts by surgeons and patients following operative treatment for kidney stones [10]. Similar to our observations, the study found that patients emphasized recovery and quality of life, while urologists concentrated on technological advances and treatment methods [10]. This suggests that patients have a strong tendency to prioritize long-term outcomes and rehabilitation over the initial treatment. Additionally, on social media, there is a greater emphasis on skin appearance and cosmetics among patients with clavicle fractures, whereas physicians maintain a focus on treatment despite often being responsible for follow-up care and rehabilitation protocols.

Another key difference observed in social media posts is the overall tone. While both surgeons and patients frequently share positive experiences or outcomes, patients are significantly more likely to post with a negative tone. One possible explanation for this lies in the concept of fault. A negative post from a surgeon may be perceived as an admission of error or an acknowledgment of a particularly challenging case, whereas a patient’s negative post is more likely to be framed as an unfortunate circumstance beyond their control.

This pattern aligns with findings from similar studies. For instance, research on social media posts from patients undergoing medial patellofemoral ligament reconstruction showed that while patients often shared positive experiences about improved function and returning to daily activities, they were also more likely to express dissatisfaction regarding surgical scars and recurrent instability [11]. Similarly, in the previously mentioned study on operatively treated kidney stones, patients frequently posted negative content, particularly about reoperations or unresolved symptoms, while not a single negatively themed post from a urologist was observed [10].

Although this difference may seem straightforward, it presents a valuable avenue for further research. Surgeons incorporating discussions about potential complications or less-than-optimal outcomes in their social media presence - without assigning blame - could help manage patient expectations and improve overall satisfaction. Providing information on topics such as nonunion rates or the timeline for returning to full activity could be especially beneficial for patients with limited medical literacy, ensuring they have a more realistic understanding of their recovery journey.

One of the most pressing concerns with the rise of social media is the spread of misinformation. While this issue often makes headlines for its impact on politics, the same principles apply to the medical field. To address this challenge, Charnock et al. developed DISCERN, a validated tool designed to help individuals assess the quality of treatment-related information [12,13]. DISCERN scores range from 1 to 5, with 1 indicating serious or extensive shortcomings and 5 representing minimal shortcomings.

Research on the accuracy of health information shared on social media using DISCERN scores has yielded mixed results. One study examining popular social media content related to common orthopedic conditions found that posts created by nonphysicians had an average DISCERN score of 2.0 - significantly lower than physician-generated posts, which averaged 3.4 [14]. Notably, physician posts also received significantly less engagement than those created by nonphysicians.

Although we did not calculate DISCERN scores for the content in our dataset, our findings align with this trend, as patient posts received more engagement than those by surgeons, based on the average number of likes and account followers. A future research objective is to assess whether there are notable differences in the accuracy of the information shared by these two groups.

Our study has several limitations. Data collection was conducted at a single point in time, capturing only a cross-sectional snapshot despite social media being a rapidly evolving landscape. As a result, the observed trends and relationships may shift over time. To mitigate this, we included four years’ worth of the most recent data available.

Additionally, we collected 417 posts from entities that were neither surgeons nor patients, including parents, medical organizations, and third-party companies. These were excluded from the analysis as they fell outside the study’s scope. However, given that these groups made up a considerable portion of the original 1,500 posts, they may play a significant role in the interaction between health information, treatment options, patients, surgeons, and social media. This represents a promising avenue for future research exploring the broader influence of social media on medicine.

Another limitation of our methodology is that we assessed only individual posts rather than full user histories. This restricted our ability to track the progression of a patient’s treatment journey or how their tone and content evolved throughout recovery. Furthermore, we could not definitively determine treatment plans for most patient posts. Given the current management trends for clavicle fractures and the lack of radiographic evidence of hardware, it can be speculated that many patients underwent nonoperative treatment. However, we were unable to confirm whether posts reflected operative or nonoperative care.

Notably, while patient posts rarely specified their treatment, surgeon-generated posts focused on operative interventions 75% of the time. This is strikingly high compared to the actual rate of surgical treatment for clavicle fractures, which is reported to be below 20% in some studies [15]. While this discrepancy does not necessarily indicate misinformation, it highlights a divide between real-world treatment patterns and their portrayal on social media. This may stem from the tendency to share more complex or unusual cases, which generate greater engagement, foster discussion, and showcase surgical expertise.

We did not assess content accuracy using a tool like DISCERN, but future research could explore the reliability of social media posts and how accuracy influences engagement. Additionally, our study focused solely on Instagram, excluding other popular platforms such as TikTok and Facebook. This limited the dataset to Instagram users within the specified time frame, and future studies should investigate trends across multiple platforms.

Despite these limitations, a key strength of this study is its relatively large sample size, with 1,083 posts analyzed. To our knowledge, this is the first and largest study to examine the portrayal of clavicle fractures on social media.

## Conclusions

Content and language used in Instagram posts about clavicle fractures differ between surgeons and patients. Surgeons tend to use technical jargon, share X-rays and educational content, adopt a positive tone, and focus on operative treatments, all while experiencing lower social media engagement. In contrast, patients emphasize the rehabilitation process and often provide a more holistic view of recovery. The interplay between health information and social media presents a promising avenue for future research, as understanding these dynamics could transform social media into a powerful tool for improving health literacy, guiding treatment decisions, and ultimately enhancing patient outcomes.

## References

[1] (2023). Number of Instagram users worldwide from 2019 to 2028 (in millions). https://www.statista.com/forecasts/1138856/instagram-users-in-the-world.

[2] Li Y, Wang X, Lin X (2018). Seeking and sharing health information on social media: a net valence model and cross-cultural comparison. Technol Forecast Soc Change.

[3] Niu Z, Willoughby J, Zhou R (2021). Associations of health literacy, social media use, and self-efficacy with health information-seeking intentions among social media users in China: cross-sectional survey. J Med Internet Res.

[4] Ropars M, Thomazeau H, Huten D (2017). Clavicle fractures. Orthop Traumatol Surg Res.

[5] Postacchini F, Gumina S, De Santis P, Albo F (2002). Epidemiology of clavicle fractures. J Shoulder Elbow Surg.

[6] Frima H, van Heijl M, Michelitsch C, van der Meijden O, Beeres FJ, Houwert RM, Sommer C (2020). Clavicle fractures in adults; current concepts. Eur J Trauma Emerg Surg.

[7] Gottfried J (2024). Americans’ social media use. Pew Res Cent.

[8] von Rüden C, Rehme-Röhrl J, Augat P, Friederichs J, Hackl S, Stuby F, Trapp O (2023). Evidence on treatment of clavicle fractures. Injury.

[9] Aydin SO, Tasargol O (2023). Spinal cord stimulation and related health information on social media: an analysis of Instagram posts. Cureus.

[10] Juliebø-Jones P, Emiliani E, Sierra A (2023). Patient perspectives on kidney stone surgery: a content analysis of Instagram posts by patients versus surgeons. Eur Urol Open Sci.

[11] Rizkalla JM, Holderread B, Botros D (2022). Patient perception of medial patellofemoral ligament reconstruction on Instagram. Proc (Bayl Univ Med Cent).

[12] Charnock D, Shepperd S, Needham G, Gann R (1999). DISCERN: an instrument for judging the quality of written consumer health information on treatment choices. J Epidemiol Community Health.

[13] Loeb S, Sengupta S, Butaney M (2019). Dissemination of misinformative and biased information about prostate cancer on YouTube. Eur Urol.

[14] Kolade O, Martinez R, Awe A, Dubin JM, Mehran N, Mulcahey MK, Tabaie S (2023). Misinformation about orthopaedic conditions on social media: analysis of TikTok and Instagram. Cureus.

[15] Kihlström C, Möller M, Lönn K, Wolf O (2017). Clavicle fractures: epidemiology, classification and treatment of 2 422 fractures in the Swedish Fracture Register; an observational study. BMC Musculoskelet Disord.

